# Molecular docking analysis of marine phytochemicals with BACE-1

**DOI:** 10.6026/973206300200151

**Published:** 2024-02-29

**Authors:** Deepak Sheokand, Annu Grewal, Pawan Kumar, Raveena Chauhan, Vandana Saini, Ajit Kumar

**Affiliations:** 1 Toxicology and Computational Biology Group, Centre for Bioinformatics, Maharshi Dayanand University, Rohtak, Haryana, India

**Keywords:** Neurodegenerative disorder, Alzheimer disease, β-amyloid, Molecular docking

## Abstract

Alzheimer's disease (AD), a debilitating neurodegenerative condition, is characterized by progressive cognitive decline brought about by the deposition of amyloid beta (Aβ) plaques in the brain initiates downstream neuronal dysfunction and death in AD pathogenesis. The β-secretase (BACE-1) enzyme plays a crucial role in generating Aβ from amyloid precursor protein (APP). Hence, we report the virtual screening of marine phytochemicals as BACE-1 inhibitors. 2583 compounds, retrieved from Comprehensive Marine Natural Product Database (CMNPD), were primarily screened for drug-likeliness and blood-brain barrier permeability using admetSAR 2.0 and *in-house* BBBper tool and resulted in a total of 635 phytochemicals, selected for further docking studies using BACE-1 as target receptor and Atabecestat as standard BACE-1 inhibitor. Seven of 635 compounds docked against BACE-1, showed better binding affinities than Atabecestat, with the red algal metabolite lactodehydrothyrsiferol showing lowest binding energy of -10.83 kcal/mol. These compounds are worth investigating further to assess their neuroprotective efficacy and pharmacokinetic properties. The study also provides a rational framework to uncover novel pharmacophores from marine sources for AD therapy acting through BACE-1 inhibition.

## Background:

Alzheimer's disease (AD) has emerged as a major public health challenge, with tens of millions afflicted globally. It is an irreversible, progressive neurodegenerative disorder leading to loss of cognitive abilities and memory. At the cellular level, AD is characterized by two key pathological hallmarks - accumulation of extracellular amyloid beta (Aβ) peptide plaques and formation of intracellular neurofibrillary tau tangles [1]. The amyloid cascade hypothesis posits that abnormal processing of amyloid precursor protein (APP) to generate soluble Aβ triggers downstream neuronal dysfunction and death central to AD pathogenesis [2]. APP processing occurs through two competing pathways. While α-secretase mediated cleavage produces non-amyloidogenic fragments, β-secretase or BACE-1 dependent cleavage results in soluble Aβ peptides that can misfold and aggregate into toxic oligomeric species [3]. BACE-1 is a trans-membrane aspartyl protease and the rate-limiting enzyme initiating amyloidogenic APP processing [4]. Compared to healthy individuals, AD patients demonstrate elevated BACE-1 expression and enzymatic activity levels in the brain [5]. Thus, therapeutic inhibition of BACE-1 could critically reduce Aβ genesis and ameliorate amyloid-induced toxicity. While small molecule BACE-1 inhibitor clinical trials have failed so far [6], natural BACE-1 blocking agents with improved safety profiles still remain promising alternatives. Marine ecosystems comprise diverse flora and fauna representing an enormous largely untapped reservoir of bioactive compounds with varied neurological effects [7]. Several marine metabolites from seaweeds, sponges and tunicates have exhibited neuroprotective, anti-inflammatory and antioxidant properties that can counter amyloid toxicity [8]. Several In-vitro studies have revealed that, Marine phytochemical, fucoidan exhibit neuroprotection [9], and improvement in spatial learning and memory [10]. Fucoxanthin is a potential neuro-therapeutic agent in future because of its spectrum of bioactivity in AD therapy. Fucoxanthin significantly decreases oxidative stress [11], inflammation [12], apoptosis [12] and leads to improvement of cognitive functions and anti-acetylcholinesterase activity [13]. Astaxanthin and fucoxanthin have been reported to display neuroprotective effect against Aβ induced toxicity and Aβ anti-aggregation properties [8]. In our study, we retrieved the physicochemical properties and structures of 2584 marine phytochemicals from the Comprehensive Marine Natural Product Database (CMNPD). 635 Marine phytochemicals were selected on the basis of drug likelihood, BBB permeability, and ADMET properties. A high-throughput virtual screening against BACE-1 (PDB id-1FKN) protein with the selected phytochemicals was performed using AutoDock v4.2.6 Software. Binding affinity and inhibition constant were evaluated using the BACE-1 inhibitor JNJ5486191 [14]. Therefore, it is of interest to report the molecular docking analysis of marine phytochemicals with BACE-1.

## Materials & Methods:

## Retrieval of phytochemicals and target protein receptor:

The structure and physicochemical property files of 2583 marine phytochemicals were retrieved from Plantae taxa under Marine Organisms category of the Comprehensive Marine Natural Product Database (CMNPD). CMNPD is a comprehensive database of about 32,000 marine chemical entities in different categories. All the retrieved phytochemicals were subjected to structural optimization using standalone Chemsketch v12.0. The target protein receptor selected for the study was BACE-1 and the 3D coordinate file of human BACE-1 was retrieved from Protein Data Bank (PDB) with PDB ID: 1FKN. The ligand and second chain of the target protein, present in retrieved file were removed and subjected to energy minimization using UCSF Chimera v1.5 to normalize the net interatomic forces acting on at each atom.

## Screening for drug likeliness and Pharmacokinetic properties:

The selected 2583 compounds were initially screened on the basis of Lipinski's rule of five for drug likelihood properties. The selected marine phytochemicals were further analysed for their bio-oral availability, Ames's mutagenicity, carcinogenicity, and hepatotoxicity using the web server admetSAR 2.0.

## Blood-brain barrier permeability prediction of selected marine phytochemicals:

The drugs acting on the CNS must be BBB-permeable. Therefore, the selected phytochemicals were screened for Blood brain permeability using three different predictions tools (CMNPD prediction, admetSAR2.0 and *in-house* developed BBBper prediction tool) and compounds observed to be BBB-permeable by all these three tools were selected for further molecular docking studies.

## Molecular docking studies:

Selected marine phytochemicals were subjected to molecular docking against human BACE-1 with PDB ID: 1FKN, using AUTODOCK v4.2.6 and Atabecestat (JNJ-54861911) as standard BACE-1 inhibitor. After the auto grid run, the appropriate map files were generated, and the grid box parameters (x = 44; y = 66; z = 70) for the binding pocket with a grid centre (13.31 -1.891 0.189) with a default spacing were used as grid parameter files (GPF) for protein receptors. The Genetic Algorithm (GA) was used to execute molecular docking and 100 independent runs were carried out using a step size of 0.2 for translation. With a mutation rate of 0.02, crossover rate of 0.8, cluster tolerance of 0.5, and external grid energy of 1000, the maximum number of gestations was set to 1000, and the maximum number of top people that automatically survived was set at 1. 2D ligand-protein interaction diagram were generated using Ligplot + v2 to analyze the polar and electrostatic interactions between ligand-protein complexes.

## Results & Discussion:

## Screening for drug likeliness and Pharmacokinetic properties:

All retrieved marine phytochemicals were screened based on Lipinski's rule, i.e., a) Molecular weight <500 Da, b) Hydrogen bond donor < 5, c) Hydrogen bond donor <10, d) logP <5 and e) Molar refractivity range 40-130. Out of 2583 marine phytochemicals, only 1480 compounds cleared Lipinski's parameters. Toxicity parameters prediction values, for the 1480 druggable compounds were retrieved from the admetSAR 2.0 web tool. Out of 1480, only 976 marine phytochemicals were selected, and the remaining compounds were predicted to be carcinogenic, Ames mutagenic, and hepatotoxic.

## Blood-brain barrier permeability prediction of selected marine phytochemicals:

A total of 756, 890 and 938 out of 1480 studied phytochemicals were observed to cross BBB as predicted by CMNPD, admetSAR and BBBper, respectively. Careful analyses resulted in 635 marine phytochemicals which were predicted to cross BBB by all the three tools and were positive hits of screening for drug likeliness and pharmacokinetic properties.

## Molecular docking studies:

**Figure 1:**
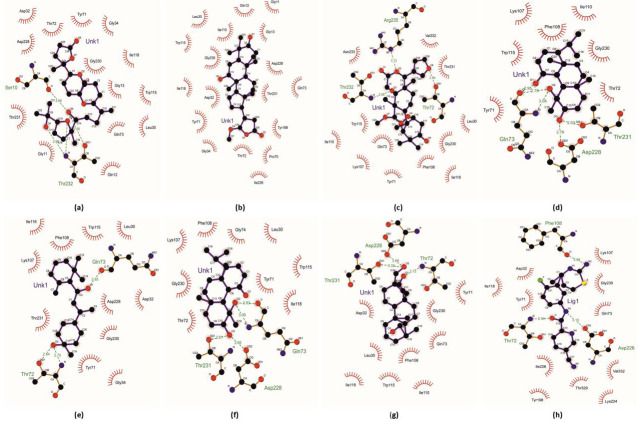
The docking site images of BACE1 protein when docked against ligands: CMNPD12563 (a); CMNPD27101 (b); CMNPD24319 (c); CMNPD2641 (d); CMNPD8860 (e); CMNPD11810 (f); CMNPD15658 (g), JNJ5486191 (h).

The molecular docking of selected marine phytochemicals against human BACE-1 with PDB ID: 1FKN and Atabecestat as standard BACE-1 inhibitor, revealed 07 compounds having better binding affinities than the standard (Table 1), the later showing binding energy of -9.61 Kcal/Mol and an inhibition constant of 5.98 µM (Figure 1; Table 2). Lactodehydrothyrsiferol (CMNPD12563), isolated from the red algae *Laurencia viridis*, was found to have highest binding affinity towards BACE-1 and formed electrostatic interactions with key residues Asp32 and Asp228 in the catalytic domain of BACE1 (Table 2, Figure 1(a)). 7-hydroxy-21β-methoxy-3-oxo-24,25,26,27-tetranortirucalla-1,14-diene-23(21)-lactone (CMNPD27101) from the mangrove plant *Xylocarpus granatum* also exhibited strong binding due to electrostatic interactions with Leu30, Asp32, Ile110 and Asp228 (Table 2, Figure 1(b)). Thaixylomolin A (CMNPD24319) from *Xylocarpus moluccensis*, (12S)-12-hydroxybromosphaerodiol (CMNPD2641) from the red algae *Sphaerococcus coronopifolius,* rogioldiol A (CMNPD8860) from *Laurencia microcladia* and bromosphaerone (CMNPD11810) from Sphaerococcus *coronopifolius* showed similar interactions with key residues in the catalytic pocket (Table 2, Figure 1(c-f)). Notably, (5β,8R,10α,13S)-16-Oxo-17-hydroxybeyera-9(11)-ene-18-al (CMNPD15658) from the mangrove species *Bruguiera rhynchopetala* also demonstrated strong binding to BACE1 comparable to the standard inhibitor JNJ5486191 (Table 1 & Figure 1(g-h).

Our in-silico docking studies identified lactodehydrothyrsiferol as a potential BACE1 inhibitor with the highest binding affinity and inhibition constant. Clausen *et al.* demonstrated the total synthesis and inhibition of protein phosphatase 2A by lactodehydrothyrsiferol *in vitro.* [15]. Importantly, the extensive biological activities of lactodehydrothyrsiferol have been reviewed, including anti-inflammatory, antitumor, antimicrobial, and neuroprotective effects [16-17]. Lactodehydrothyrsiferol was also found to inhibit acetylcholinesterase activity, suggesting cognitive enhancement [18]. Its unique polyhalogenated structure likely confers potent bioactivity. Our docking studies also identified 7-hydroxy-21β-methoxy-3-oxo-24,25,26,27-tetranortirucalla-1,14-diene-23(21)-lactone (CMNPD27101) from *X. granatum* as a potential BACE1 inhibitor. Wu *et al.* isolated novel limonoids including CMNPD27101 from *X. granatum* and evaluated their antifeedant activity [19]. In antifeedant assays against armyworm larvae, CMNPD27101 exhibited significant activity, confirming the bioactivity of this compound in *vivo*. *X. granatum* extracts containing limonoids like CMNPD27101 have also shown antiviral, antioxidant, and neuroprotective effects [20- 21].

Additionally, CMNPD27101 belongs to a class of tirucallane triterpenoids which have demonstrated diverse bioactivities including anticancer, anti-inflammatory, and antimicrobial properties Limonoid thaixylomolin A (CMNPD24319) from *X. moluccensis* was also identified as a potential BACE1 inhibitor. Importantly, Sarker *et al.* demonstrated that extracts of *X. moluccensis* containing compounds like thaixylomolin A improved learning and memory in rats [23]. This provides in *vivo* evidence for the neuroprotective effects of thaixylomolin A. Other studies have also shown antioxidant, anti-inflammatory, and acetylcholinesterase inhibitory activities of *X. moluccensis* extracts and limonoids [24], further supporting their bioactivity. Additionally, our docking predicted (12S)-12-hydroxybromosphaerodiol (CMNPD2641) from the red alga *S. coronopifolius* as a potential BACE1 inhibitor. Others studies also demonstrated that CMNPD2641 significantly attenuated 6-OHDA-induced toxicity in SH-SY5Y neuronal cells *in vitro.*, indicating neuroprotective affect. Other bromoditerpenes from *S. coronopifolius* also exhibited antioxidant and antimicrobial activities **in vitro.*.* Overall, the in *vivo* and *in vitro.* studies on these marine phytochemicals provide evidence to support their potential bioactivity as BACE1 inhibitors for Alzheimer's therapeutics. Our docking studies indicate the potential of these seven marine phytochemicals as novel BACE1 inhibitors for Alzheimer's disease therapeutics and further *in vitro.* and in *vivo* studies are warranted to evaluate their neuroprotective efficacy. [22].

## Conclusion:

There is a lack of drug availability for different stages of AD progression and modern anti-acetylcholinesterase inhibitors can only alleviate symptoms in the early or middle stages of the disease but fails to work in later stages of AD. Inhibition of β-amyloid deposition can prevent neuronal death. Potential inhibitors of BACE-1 enzyme show reduced β-amyloid deposition in synaptic cleft. Our study indicates the potential of seven marine phytochemicals as efficient BACE1 inhibitors for developing AD's therapeutics.

## Figures and Tables

**Table 1:** The marine phytochemicals showing better binding affinities (lower binding energies) than the standard Atabecestat (JNJ5486191), when docked against BACE1 protein

**Compound ID**	**Binding Energy (kcal/mol)**	**Name**	**Plant spp.**
CMNPD12563	-10.83	Lactodehydrothyrsiferol	*Laurencia viridis*
CMNPD27101	-10.06	7-Hydroxy-21β-methoxy-3-oxo-24,25,26,27-tetranortirucalla-1,14-diene-23(21)-lac-tone	*Xylocarpus granatum*
CMNPD24319	-9.82	Thaixylomolin A	*Xylocarpus moluccensis*
CMNPD2641	-9.77	(12S)-12-Hydroxybromosphaerodiol	*Sphaerococcus coronopifolius*
CMNPD8860	-9.75	Rogioldiol A	*Laurencia microcladia*
CMNPD11810	-9.71	Bromosphaerone	*Sphaerococcus coronopifolius*
CMNPD15658	-9.66	(5β,8R,10α,13S)-16-Oxo-17-hydroxybeyera-9(11)-ene-18-al	*Bruguiera rhynchopetala*
Atabecestat (JNJ5486191)	-9.61	NA	NA

**Table 2:** H-bond and hydrophobic interactions of selected phytochemicals when docked against BACE1 protein

**Compound ID**	**H bond**	**Hydrophobic interaction**
CMNPD12563	Ser10, Thr232	Gly11, Gln12, Gly13, Leu30, Asp32, Gly39, Tyr71, Thr72, Gln73, Trp115, Ile118, Asp228, Gly230, Thr231
CMNPD27101	--	Gly11, Gln12, Gly13, Leu30, Asp32, Gly34, Pro70, Tyr71, Thr72, Gln73, Ile110, Trp115, Ile118, Tyr198, Ile226, Thr231, Asp228, Gly230
CMNPD24319	Thr72, Thr232, Arg235	Leu30, Tyr71, Gln73, Lys107, Phe108, Ile110, Trp115, Ile118, Gly230, Thr231, Asn233, Val332
CMNPD2641	Gln73, Asp228, Thr231	Tyr71, Thr72, Lys107, Phe108, Ile110, Trp115, Gly230
CMNPD8860	Thr72, Gln73,	Lys10, Thr23, Leu30, Asp32, Gly34, Tyr71, Phe108, Trp115, Ile118, Gly230,
CMNPD11810	Gln73, Asp228, Thr231	Leu30, Tyr71, Thr72, Gly74, Lys107, Phe108, Trp115, Ile118, Gly230
CMNPD15658	Thr72, Asp228, Thr231	Leu30, Asp32, Tyr71, Gln73, Phe108, Ile110, Trp115, Ile118
Atabecestat (JNJ5486191)	Thr72, Phe108, Asp228,	Asp32, Gln73, Tyr71, Lys107, Ile118, Tyr198, Lys224, Ile226, Gly230, Thr329, Val332
